# Characterizing the D-Amino Acid Position in Peptide Epimers by Using Higher-Energy Collisional Dissociation Tandem Mass Spectrometry: A Case Study of Liraglutide

**DOI:** 10.3390/ijms25031379

**Published:** 2024-01-23

**Authors:** Yuan-Chih Chen, Hsin-Yi Wu, Lung-Cheng Lin, Chih-Wei Chang, Pao-Chi Liao

**Affiliations:** 1 Department of Environmental and Occupational Health, College of Medicine, National Cheng Kung University, Tainan 704, Taiwan; 2 Instrumentation Center, National Taiwan University, Taipei 106, Taiwan; 3 ScinoPharm Taiwan, Ltd., Tainan 741, Taiwan

**Keywords:** chirality, high-resolution mass spectrometry, D-amino acid-containing peptide, higher-energy collisional dissociation

## Abstract

D-amino acid-containing peptides (DAACPs) occur in biological and artificial environments. Since the importance of DAACPs has been recognized, various mass spectrometry-based analytical approaches have been developed. However, the capability of higher-energy collisional dissociation (HCD) fragmentation to characterize DAACP sites has not been evaluated. In this study, we compared the normalized spectra intensity under different conditions of HCD and used liraglutide along with its DAACPs as examples. Our results indicated that the difference in the intensity of y ions between DAACPs and all-L liraglutide could not only distinguish them but also localize the sites of D-amino acids in the DAACPs. Our data demonstrate the potential of using HCD for the site characterization of DAACPs, which may have great impact in biological studies and peptide drug development.

## 1. Introduction

In nature, most amino acids contained in peptides and proteins are L-amino acids or achiral. However, the epimers of these common amino acids, D-amino acids, also exist in some biological [1] or artificial peptides [2]. These D-amino acid-containing peptides (DAACPs) have been reported to have specific biological functions or influence the efficacy of pharmaceuticals. For example, more than 30 DAACPs have been identified in animals [3], 4 of which occur in the human body [4,5,6,7] causing key effects in the pathogenesis of some diseases, such as Alzheimer’s disease [8]. A series of aging-related diseases were also reported to associate with DAACPs following racemization in proteins [1]. Regarding artificial peptides, D-amino acids were reported to enhance the biostability of peptide drugs in the human body, since D-amino acids usually change the secondary structure of peptide drugs and interfere with the interaction between peptide drugs and their receptors [9,10]. On the contrary, DAACPs could also be accidentally synthesized and considered as undesired impurities during solid-phase peptide drug synthesis (SPPS) [11,12]. The inescapable probability of racemization during SPPS (approximately 0.4% or less per synthesis cycle) [13] indicated that the identification of DAACP impurities in peptide drug products is required and imposes an urgent quality control of biological therapeutics [14,15,16].

Several analytical strategies have been developed for identifying DAACP features. Enzyme digestion, such as by using aminopeptidase M, can digest peptides starting from the N-terminus and stop when a D-amino acid is encountered, revealing the location of the modification [17,18,19]. Chromatographic separation with a reverse-phase column has been used as a tool for studying most isomeric peptides [20,21,22]. Capillary electrophoresis (CE) has been proposed as another tool to separate isomeric peptides [23], and it is suggested to be coupled with MS when dealing with complex mixtures. Although mass spectrometry (MS)-based approaches have been well established for analyzing proteins and peptides, it is still challenging to apply them for DAACP analysis, since the isomerization modification dose not result in any change in peptide mass. However, MS requires only trace amounts of samples and can be coupled with various separation systems such as LC, CE and ion mobility spectrometry, which makes MS a core technique for analyzing DAAPCs. Moreover, characterizing the specific sites of isomerization is critical for it is not practical to synthesize all possible DAACPs to verify the retention times. It was reported that combining enzymatic screening, chiral analysis, and liquid chromatography mass spectrometry analysis and confirmation can discover novel DAAPCs [18].

Ion mobility spectrometry coupled with mass spectrometry (IM-MS) has been demonstrated as a promising tool for characterizing peptide epimers [24,25,26,27]. In addition to the separation of precursor ions of DAACPs, the analysis of the ion mobility of epimeric peptide fragment ions could also characterize the specific sites of D-amino acids in DAACPs [28]. In addition, a variety of unique fragmentation techniques, including MALDI-TOF/TOF [21,29], electron capture dissociation (ECD) [30], radical-directed dissociation (RDD) [31] and electron transfer dissociation (ETD) [32], have been reported to distinguish DAACPs from normal peptides. Collision-induced dissociation (CID), on the other hand, is a common type of tandem mass spectrometry technique. According to the design of the collision cell and fragmentation mechanism, two types of CID, ion trap and low-energy beam, have been applied for peptide sequence identification. However, both types of CID have been reported to not always work well for discriminating DAACPs [30,31,33]. Although CID has poor capability in DAACP characterization, it is indicated that CID could sometimes provide site information within a few residues, which narrows the possibility of chirality in DAACPs [33]. One of the ion trap CID techniques, higher-energy collisional dissociation (HCD), is gradually becoming widely used and provides fragment-rich spectra, since it avoids the low mass cutoff problem intrinsic to the traditional ion trap CID. It was found that the fragment intensity in HCD results is different between L- and D-peptides [34]; however, the use of HCD for site localization has not been evaluated. Here, we attempted to evaluate the capability of HCD to characterize DAACPs, particularly, to localize D-residues. Liraglutide, an analog of glucagon-like peptide-1 (GLP-1), approved in 2010 [35] and sold under the name of Victoza, was used as model DAACP. Fourteen DAACP and all-L liraglutide standards were systematically analyzed using high-resolution mass spectrometry (HRMS) at various HCD collision energies. The tandem mass spectrometry (MS/MS) data of the DAACPs and all-L liraglutide were processed using RStudio version 2023.12.0 and R version 4.3.2. The MS/MS data characterizing the DAACPs were comprehensively investigated.

## 2. Results

### 2.1. Analytical Procedure

**Figure 1:**
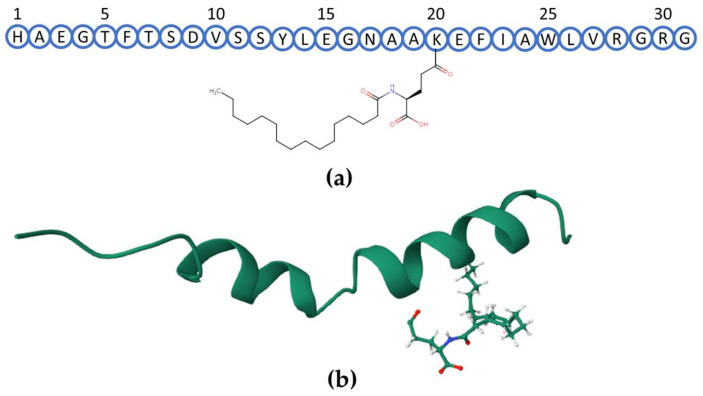
Sequence and schematic structure of liraglutide. (**a**) Sequence of liraglutide, including the side chain on Lys^20^. (**b**) Three-dimensional view of liraglutide; visualizing software: RCSB website (PDB DOI: https://doi.org/10.2210/pdb4APD/pdb (accessed on 21 January 2024)).

Liraglutide contains thirty-one amino acids (1-HAEGTFTSDVSSYLEGNAAKEFIAWLVRGRG-31) and a side chain of palmitoyl glutamic acid linked to lysine 20 (Figure 1). Fourteen DAACP standards of liraglutide with D-amino acids in different sites, i.e., His^1^, Ala^2^, Phe^6^, Asp^9^, Ser^11^, Ser^12^, Tyr^13^, Glu^15^, Gln^17^, Ala^18^, Ala^19^, Lys^20^, Glu^21^, Arg^30^, were prepared for a systematic investigation.

**Figure 2:**
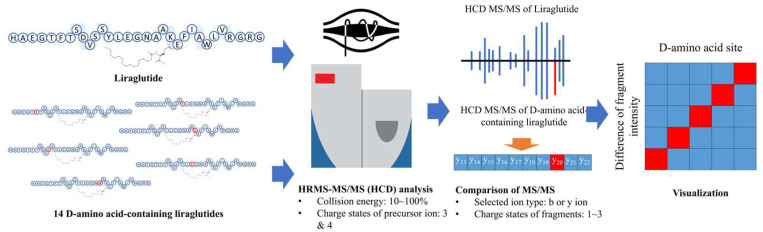
Study design used to systematically evaluate the capability of higher-energy collisional dissociation (HCD) in D-amino acid-containing peptide (DAACP) characterization.

A systematic evaluation of the impact of HCD on DAACP characterization is illustrated in Figure 2. Standards of liraglutide (all-L) and its 14 DAACP counterparts were analyzed by high-resolution tandem mass spectrometry using HCD fragmentation (HR-HCD-MS/MS). The triply and quadruply charged ions of liraglutide/DAACPs were observed in full-scan spectra and selected as precursor ions for the following MS/MS analysis. A series of normalized collision energies (NCEs), between 10 and 100%, were applied to understand the chirality-related characteristics of DAACPs in HCD (Figures S1 and S2). Analysis using different NCEs was repeated 20 times for each peptide to evaluate the statistical significance of the observed difference. The raw MS/MS spectra were processed by in-house software, which generated comparison results between fragments from liraglutide (all-L) and each DAACP. Differentiations in fragment ions, including b or y ions and charge states, were evaluated, and the relationship between fragment ion intensity and site of D-amino acid was visualized in a heatmap.

### 2.2. Comparison between the MS/MS Spectra of Liraglutide and DAACPs

**Figure 3:**
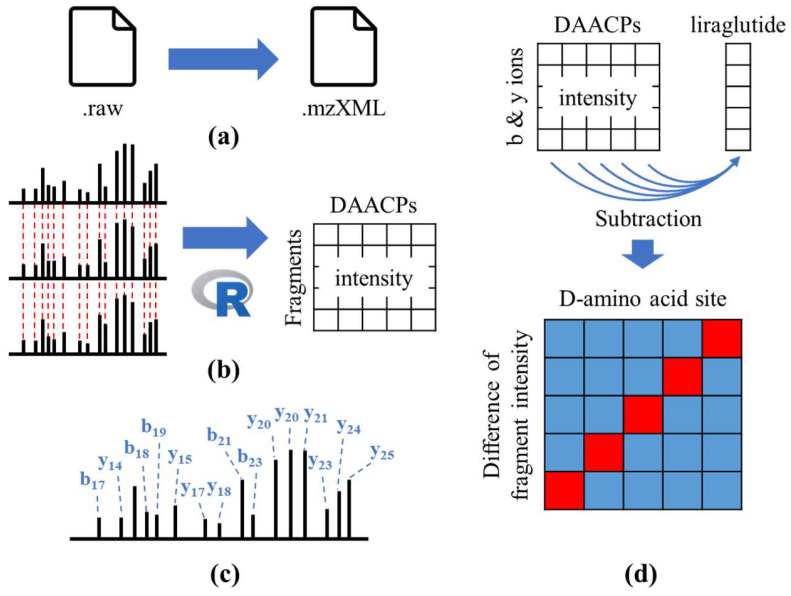
Workflow used to process the high-resolution mass spectrometry (HRMS) data of liraglutide and the 14 DAACPs. (**a**) Raw data were converted to .mzXML files, and (**b**) their tandem mass spectrometry (MS/MS) fragments were aligned by R language-based in-house software. (**c**) Fragments were annotated by matching to theoretical b and y ions. (**d**) Intensity differences for each fragment from the DAACPs and all-L liraglutide were calculated and are illustrated. If the fragment intensity of DAACP is higher than that of all-L liraglutide, the color will be red. Otherwise, the color will be blue.

The MS/MS spectra of liraglutide from two precursor ions (*m*/*z* 1250.6561 Da, 3+; *m*/*z* 938.2439 Da, 4+) obtained using multiple collision energies are illustrated in Figures S1 and S2. Complete fragments of triply and quadruply charged precursor ions could be observed with NCEs of 25~45% and 15~40%, respectively. The detailed workflow of the data processing is illustrated in Figure 3. The raw HRMS data of liraglutide and the 14 DAACPs were first converted into mzXML files by msConvert software version 3 [36]. R language-based in-house software with the readMzXmlData package was applied to generate an MS/MS peak table for the 14 DAACPs, including fragment detection and alignment. For fragment detection, the in-house software averaged the *m*/*z* values and intensities of each fragment in all MS/MS scans from each 2 min segment in the HRMS data. After the fragments from the MS/MS data were chosen, their spectra were aligned with other spectra with *m*/*z* tolerance <10 ppm and exported in a peak table. The fragments listed in the peak table were annotated by matching their m/z values with theoretical *m*/*z* values of b and y ions, which were calculated by the proteomics toolkit Fragment Ion Calculator (Institute for Systems Biology, Seattle, WA, USA), with tolerance <10 ppm. The intensity of each matched fragment was normalized by the sum of all matched fragments. The differences in fragment intensities between liraglutide and the 14 DAACPs were calculated based on the intensity of each fragment obtained for liraglutide and the 14 DAACPs. A visualization plot was created using Origin software version 2021 (9.8) (OrginLab Corporation, Northampton, MA, USA) to show the relationship between the D-amino acid sites and the differences in fragment intensity.

**Figure 4:**
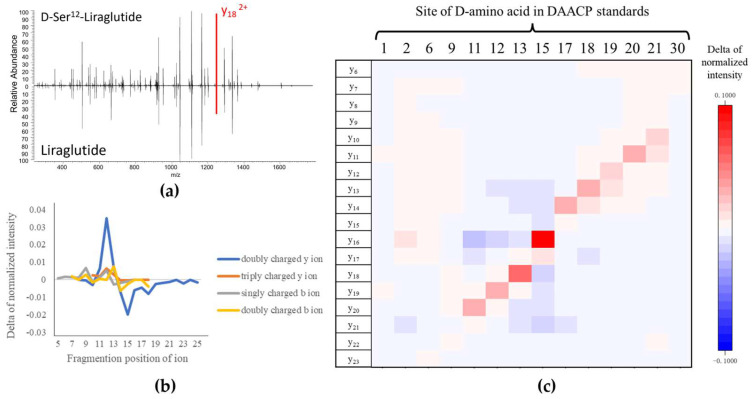
Differences in normalized fragment ion intensities between liraglutide and DAACPs. (**a**) MS/MS spectra of liraglutide and D-Ser^12^-liraglutide (*m*/*z* 938.2439 Da, 4+). The intensity of doubly charged y_18_ ions significantly differs in the two spectra. (**b**) Relationship between normalized fragment ion intensities and types of ions; only doubly charged y ions significantly differed. The MS/MS spectra of D-Ser^12^-liraglutide (*m*/*z* 938.2439 Da, 4+) are shown as an example. (**c**) Relationship between the difference in the normalized doubly charged y ion intensity and the site of the D-amino acid. The relatively intense fragment ions correspond to D-amino acids located in positions between 11 and 21. Data were generated by using normalized collision energy (NCE) of 20%.

A comparison between the MS/MS spectra of liraglutide (all-L) and D-Ser^12^-liraglutide (*m*/*z* 938.2439 Da, 4+) is provided in Figure 4a, and other comparisons are shown in Figure S3. Fragment ions with obvious differences could be observed in 9 of the 14 MS/MS spectra (Ser^11^-, Ser^12^-, Tyr^13^-, Glu^15^-, Gln^17^-, Ala^18^-, Ala^19^-, Lys^20^-, Glu^21^-DAACPs, labeled as red peaks). The D-amino acids of these nine DAACPs were located in the middle of the sequence of liraglutide (11~21). The differences in fragment ion intensity were also confirmed by the Student’s *t*-test (Table S1). The fragment ions in the nine DAACPs showed significant differences (*p* < 0.05) in the MS/MS spectra of liraglutide and the DAACPs under NCEs of 15~30% in HCD. Noteworthily, all the fragments with significant differences were doubly charged y ions. To further address how the charge can impact the characterization of D-amino acid sites in a sequence, the intensity differences for four types of the observed fragment ions, y ions (2+, 3+) and b ions (1+, 2+), generated from D-Ser^12^-liraglutide, were evaluated. We found that doubly charged y ions yielded from quadruply charged precursor ions showed the greatest differences (Figure 4b). In addition, doubly charged y ions from the other eight DAACPs also showed significant differences compared to those from liraglutide (Figure S4).

The relationship between the difference in normalized fragment ion intensity and the sites of D-amino acids in the 14 DAACPs is illustrated in Figure 4c. Each row indicates a doubly charged y ion, and each column indicates a different DAACP with a D-amino acid in a specific site. The differences in the normalized intensity of y ions from all-L liraglutide and D-Ser^12^-liraglutide (∆I) are presented in a heatmap. Significant changes in ∆I near the modification sites corresponding to amino acids 11–21 were noted. Although the doubly charged y ions from quadruply charged precursor ions contained valuable information for distinguishing the DAACPs and localizing the sites of the D-amino acids, the fragments from the triply charged precursor ions were not able to distinguish the modified sites in the DAACPs (Figure S5).

**Figure 5:**
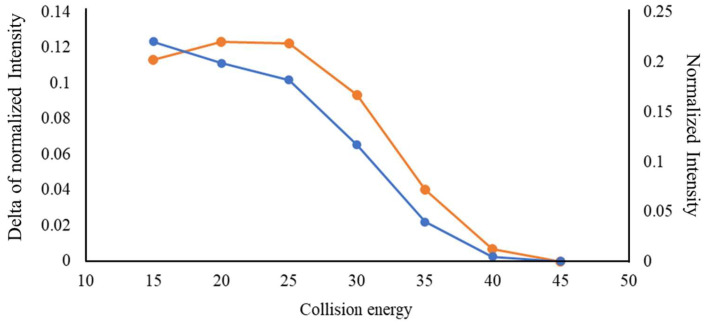
Raw intensity and delta of normalized intensity (∆I) for the y_16_^2+^ ion from D-Glu^15^-liraglutide (*m*/*z* 938.2439 Da, 4+), generated by using a NCE of 10~50%.

It was noted that the NCE also significantly impacted the intensity difference between doubly charged y ions associated with each D-amino acid in the DAACPs and the corresponding sites in all-L liraglutide. Figure 5 illustrates the raw intensity of y_16_^2+^ from D-Glu^15^-liraglutide and its delta of normalized intensity (∆I) for NCEs from 15 to 50%. The analysis results with NCE of 10% and of 50~100% were not included because y_16_^2+^ ions were not detected under these conditions. A significant ∆I was observed when the NCE was lower than 40%. However, ∆I was larger for a NCE of 15% than for a NCE of 20%, even though the raw intensity with a NCE of 15% was lower than that obtained with a NCE of 20%. This observation revealed a non-linear relationship between the intensity of the fragment ions and the intensity variations attributed to the D-amino acids in DAACPs.

### 2.3. Linearity between Fragment Ion Intensity and Proportion of DAACPs

**Figure 6:**
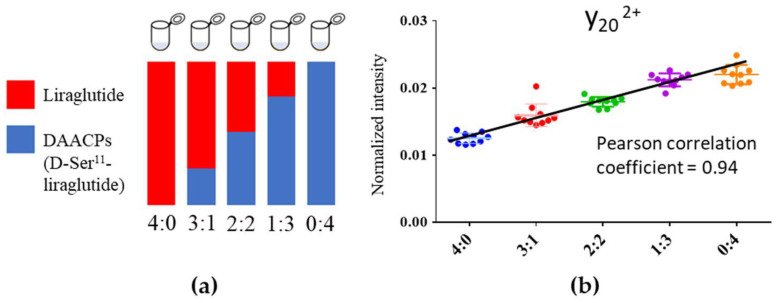
Linearity of the relationship between the ratio of liraglutide/DAACP and fragment ion intensity. (**a**) Five ratios (4:0; 3:1; 1:1; 1:3; 0:4) of liraglutide/DAACP were prepared for evaluating linearity. (**b**) The intensity of the fragment ion y_20_^2+^ increases with the amount of DAACP in the standard mixture.

In order to investigate how the signal intensity of the key fragment ion that can identify a D-amino acid site will change according to the amount of DAACP in the sample, various ratios of all-L liraglutide to D-Ser^11^ DAACP were analyzed. As shown in Figure 6, five mixtures with different all-L liraglutide-to-D-Ser^11^ liraglutide concentration ratios (4:0; 3:1; 1:1; 1:3; 0:4; the purity difference between all-L to D-Ser^11^ liraglutide was normalized) were prepared, and the intensity of the signature fragment ion y_20_^2+^ was measured. The intensity increased along with the concentration ratio and showed a high correlation (0.94, Figure 6b). Intensity differences using mixtures of all-L liraglutide and other D-standards were also evaluated by the Student’s *t*-test, as illustrated in Figure 6b. All four concentrations of D-Ser^11^ DAACP showed significant differences compared to all-L liraglutide, revealing the potential of the HCD fragmentation for the quantitation of DAACPs in all-L peptide–DAACP mixtures.

## 3. Discussion

### 3.1. Capability of HCD to Characterize DAACPs

It was reported that CID is not suitable for discriminating DAACP epimers [30,31,33]. Our data indicated that HCD has great potential to distinguish DAACPs from all-L peptides and provide modified-site information regarding D-amino acids. Specific conditions were needed for HCD-based DAACP characterization. First, compared to the natural DAACPs investigated by previous studies [18,28], liraglutide, used in this study, is a relatively long peptide of 31 amino acids. Since only the D-amino acid located in the middle of the peptide sequence could be significantly characterized by HCD analysis, the signature fragment ion intensity pattern of the short peptide might show similar features in all-L and DAACP peptides. Second, the ion types of precursor and fragment ions selected could also significantly affect whether HCD can distinguish DAACPs from normal peptides. The example of liraglutide indicated that only doubly charged y ions from quadruply charged precursor ions showed a difference in fragment ion intensity related to the site of D-amino acids. Third, the selection of NCE plays an important role in HCD-based DAACP characterization. As observed for the fragment ion intensity and the difference in normalized intensity of fragment ions for NCE of 15% and 25%, the optimized NCE with the most abundant fragments may not generate the greatest difference for distinguishing DAACPs.

### 3.2. Possible Mechanism Leading to the Higher Ion Intensity at the Site-Related Fragment

The connection between significantly different doubly charged y ion intensities and D-amino acid sites in DAACPs in specific experimental conditions indicated that an unknown chirality-related feature might occur in the mechanism underlying HCD-based DAACP fragmentation. Unfortunately, due to the required extremely huge amount of calculation, it is difficult to directly simulate the HCD fragmentation of liraglutide and DAACPs [37,38]. However, several reported publications investigated the influences of D-amino acids on the fragmentation of small cyclic peptides using two amino acids [39,40,41]. One of the publications [41] reported that the intensity of the CO loss fragment, which utilizes the same fragmentation site as the y ion, increased in “LD” cyclic peptides compared to “LL” cyclic peptides. The simulated mechanism indicated that the differentiation in CO loss fragment intensity was attributed to the charge dissymmetry, which was caused by the proton located on the side chain. The longer sequence of liraglutide with more three-dimensional effects might enhance the charge dissymmetry effect and generate specific characteristics in the HCD data of liraglutide and DAACPs. With the increase in computer performance and the development of efficient and accurate fragmentation simulation approaches, the understanding of the mechanism of DAACP fragmentation is expected to be realized soon.

The HCD fragmentation results of DAACPs and all-L liraglutide indicated that only the DAACPs with the D-amino acid located in the middle of the peptide sequence showed a significant change in fragment ion intensity. One of the possible causes of this finding is that the D-amino acid located in the middle of the DAACP sequence might significantly transform the secondary structure of the peptide. The significant change in peptide stereoconfiguration might further enhance the effect of the chirality-related mechanism of HCD fragmentation. Since the secondary structures of the 14 DAACPs were not determined in previous publications and available software for peptide secondary structure simulation, such as AlphaFold2 [42] and PEP-FOLD [43], does not support the prediction of the peptides with D-amino acids, the relationship between the secondary structure of DAACPs and the mechanisms underlying the change in fragment ion intensity cannot be proved, currently. To uncover the mechanism and the characteristics of HCD-based DAACP fragmentation, further investigation and the development of DAACP-related peptide secondary structure and fragmentation simulation software are urgently needed.

## 4. Materials and Methods

### 4.1. Chemicals and Sample Preparation

Standards of liraglutide and the 14 DAACPs were synthesized by ScinoPharm Taiwan, Ltd. (Tainan, Taiwan). The purity of normal liraglutide was approximately 95%, and the purity of the DAACP standards was approximately 35%. LC–MS-grade methanol (MeOH, purity, ≥99.9%) was purchased from Merck (Darmstadt, Germany). HPLC-grade acetonitrile (ACN, purity, ≥99.9%) was purchased from J.T. Baker (Phillipsburg, NJ, USA). HPLC-grade formic acid (FA, 98–100%) was purchased from Sigma-Aldrich (St. Louis, MO, USA).

Standards of liraglutide and the 14 DAACPs were first prepared in 1 mg/mL of standard solutions with a 50% ACN/water solution. The standard solutions were further diluted to 1 μg/mL samples and spiked with formic acid to a concentration of 0.1% for HRMS analysis.

### 4.2. HRMS Analysis

HRMS analyses were performed using an Orbitrap Q Exactive Plus Hybrid mass spectrometer (Thermo Fisher Scientific, Bremen, Germany). The prepared liraglutide and 14 DAACP samples were injected into the HR mass spectrometer with a syringe (500 µL Gastight Syringe Model 1750, Hamilton). The injection rate was set at 180 μL/hr. Between successive injections of the standards, the syringe was cleaned with 500 µL of 50% ACN in a series of three wash cycles.

HRMS data of the injected standards were acquired with a 40 min analytical run in positive mode. A 2 min full scan analysis was first performed, followed by followed by 19 HCD analyses with collision energies from 10% to 100% (a 5% increase in collision energy was applied each time). The HCD analyses were acquired in parallel reaction monitoring (PRM) mode with two set *m*/*z* values of precursor ions and 3 and 4 charges of the [M+H]+ ions of liraglutide (*m*/*z* = 1250.6561 and 938.2439). The specific parameters employed for the HRMS analysis were as follows: for the full scan analysis, the *m*/*z* range was set between 200 and 2000 *m*/*z*, with a resolution of 70,000 FWHM, automatic gain control (AGC) set at 3 × 10^6^, and a maximum injection time of 200 ms. For the MS/MS analysis, the *m*/*z* range spanned from 200 to 2000 *m*/*z*, with a resolution of 17,500 FWHM, AGC set at 1 × 10^5^, an isolation window of 1.6 *m*/*z*, and a maximum injection time of 50 ms.

## 5. Conclusions

In this study, the capability of HCD to characterize DAACPs was investigated. The analysis results indicated that HCD fragmentation could distinguish all-L liraglutide and DAACPs based on the difference in intensity of a specific fragment ion and the location of the D-amino acid site on the DAACPs. These significant intensity differences were observed in the doubly charged y ions from quadruply charged precursor ions, with relatively low collision energies (HCD of 15~20%) and the D-amino acid located in the middle of the DAACPs. Our data shed light on the potential of HCD for D-amino acid site characterization in DAACPs. To further validate and investigate the relationship between DAACPs and HCD fragmentation, more HCD-based DAACP analyses and the investigation of the DAACP fragmentation mechanisms in HCD are needed.

## Data Availability

Data are contained within the article and the Supplementary Materials.

## Supplementary Materials

The following supporting information can be downloaded at https://www.mdpi.com/article/10.3390/ijms25031379/s1.
