# Supplementary material for: Characterizing the D-Amino Acid Position in Peptide Epimers by Using Higher-Energy Collisional Dissociation Tandem Mass Spectrometry: A Case Study of Liraglutide
Source: Int J Mol Sci. 2024 Jan 23;25(3):1379. doi: 10.3390/ijms25031379 (PMC10855602; doi:10.3390/ijms25031379)

Supporting Information

Characterizing the D-Amino Acid Position in Peptide Epimers by using Higher-Energy Collisional Dissociation Tandem Mass Spectrometry: a Case Study of Liraglutide

Yuan-Chih Chen [1,^], Hsin-Yi Wu [2,^], Lung-Cheng Lin [3], Chih-Wei Chang [1], and Pao-Chi Liao [1,*]

1. Department of Environmental and Occupational Health, College of Medicine, National Cheng Kung University, Tainan 704, Taiwan
2. Instrumentation Center, National Taiwan University, Taipei 106, Taiwan
3. ScinoPharm Taiwan, Ltd., Tainan 741, Taiwan

[*]Correspondence:

Dr. Pao-Chi Liao

Department of Environmental and Occupational Health

National Cheng Kung University College of Medicine

138 Sheng-Li Road, Tainan 70428, Taiwan

TEL: 886-6-2353535 ext 5566, FAX: 886-6-2743748

E-mail: liaopc@mail.ncku.edu.tw

Table S1. Student's T-test results of the differences of fragment ion intensity (D-amino acid corresponding doubly charged y ion).

Student's T-test p value (-log$_{10}$)	Collision energy (HCD%)					
	15%	20%	25%	30%	35%	40%
D-His[1]-Liraglutide	N.D.	N.D.	N.D.	N.D.	N.D.	N.D.
D-Ala[2]-Liraglutide	N.D.	N.D.	N.D.	N.D.	N.D.	N.D.
D-Phe[6]-Liraglutide	N.D.	N.D.	N.D.	N.D.	N.D.	N.D.
D-Asp[9]-Liraglutide	13.39	9.92	10.11	3.78	N.D.	N.D.
D-Ser[11]-Liraglutide	42.39	34.81	35.88	28.73	15.72	0.00
D-Ser[12]-Liraglutide	35.77	42.13	30.37	35.44	27.20	0.00
D-Tyr[13]-Liraglutide	43.53	51.86	46.76	49.37	36.15	2.92
D-Glu[15]-Liraglutide	41.41	50.64	45.36	41.95	27.48	14.00
D-Gln[17]-Liraglutide	20.26	24.44	24.77	22.50	12.25	0.64
D-Ala[18]-Liraglutide	64.03	63.85	52.31	38.82	0.00	0.00
D-Ala[19]-Liraglutide	33.40	32.73	34.81	36.23	24.47	0.00
D-Lys[20]-Liraglutide	38.36	36.26	37.00	29.94	20.96	9.94
D-Glu[21]-Liraglutide	27.24	35.02	31.61	26.39	15.63	4.51
D-Arg[30]-Liraglutide	N.D.	N.D.	N.D.	N.D.	N.D.	N.D.

N.D.: Not detected

Figure S1. MS/MS spectra of liraglutide from triply charged precursor ion. MS/MS spectra of HCD% < 20% and > 45 are ignored since no fragments appeared in spectra. a. HCD=20%, b. HCD=25%, c. HCD=30%, d. HCD=35%, e. HCD=40%, f. HCD=45%.

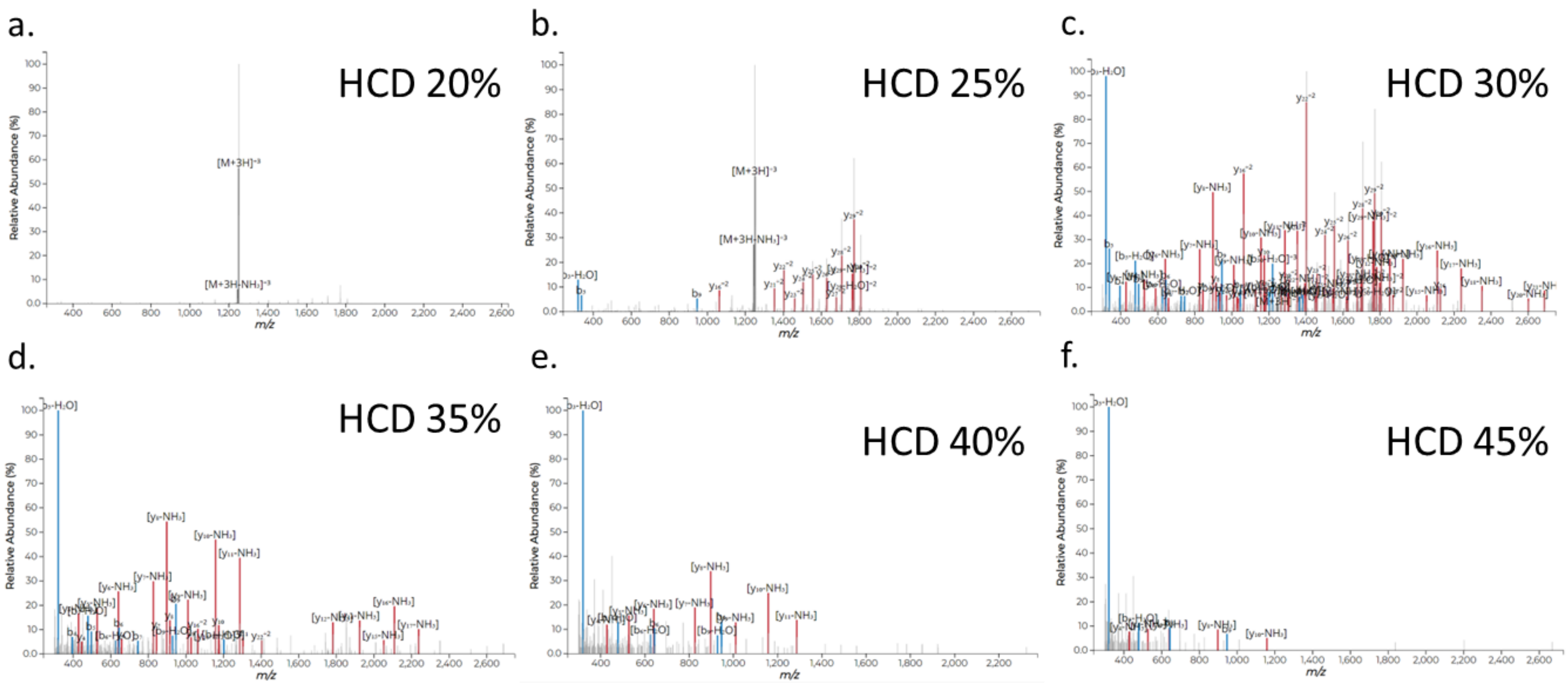

Figure S2. MS/MS spectra of liraglutide from quadruply charged precursor ion. MS/MS spectra of HCD% > 55 are ignored since no fragments appeared in spectra. a. HCD=10%, b. HCD=15%, c. HCD=20%, d. HCD=25%, e. HCD=30%, f. HCD=35%, g. HCD=40%, h. HCD=45%, i. HCD=50%, j. HCD=55%.

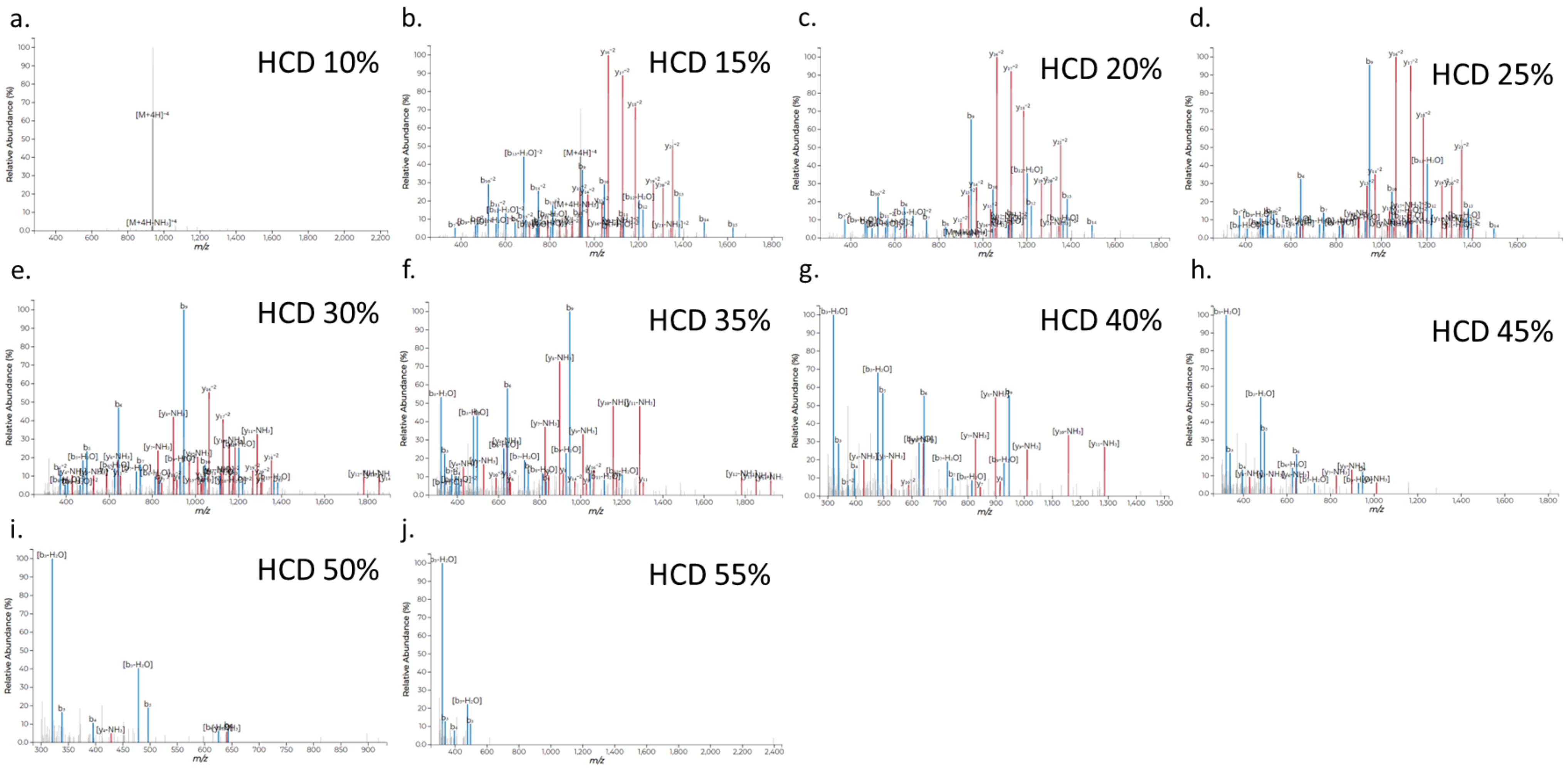

Figure S3. Fragments between liraglutide and 14 DAACPs. a. D-His[1]-Liraglutide, b. D-Ala[2]-Liraglutide, c. D-Phe[6]-Liraglutide, d. D-Asp[9]-Liraglutide, e. D-Ser[11]-Liraglutide, f. D-Tyr[13]-Liraglutide, g. D-Glu[15]-Liraglutide, h. D-Gln[17]-Liraglutide, i. D-Ala[18]-Liraglutide, j. D-Ala[19]-Liraglutide, k. D-Lys[20]-Liraglutide, l. D-Glu[21]-Liraglutide, m. D-Arg[30]-Liraglutide.

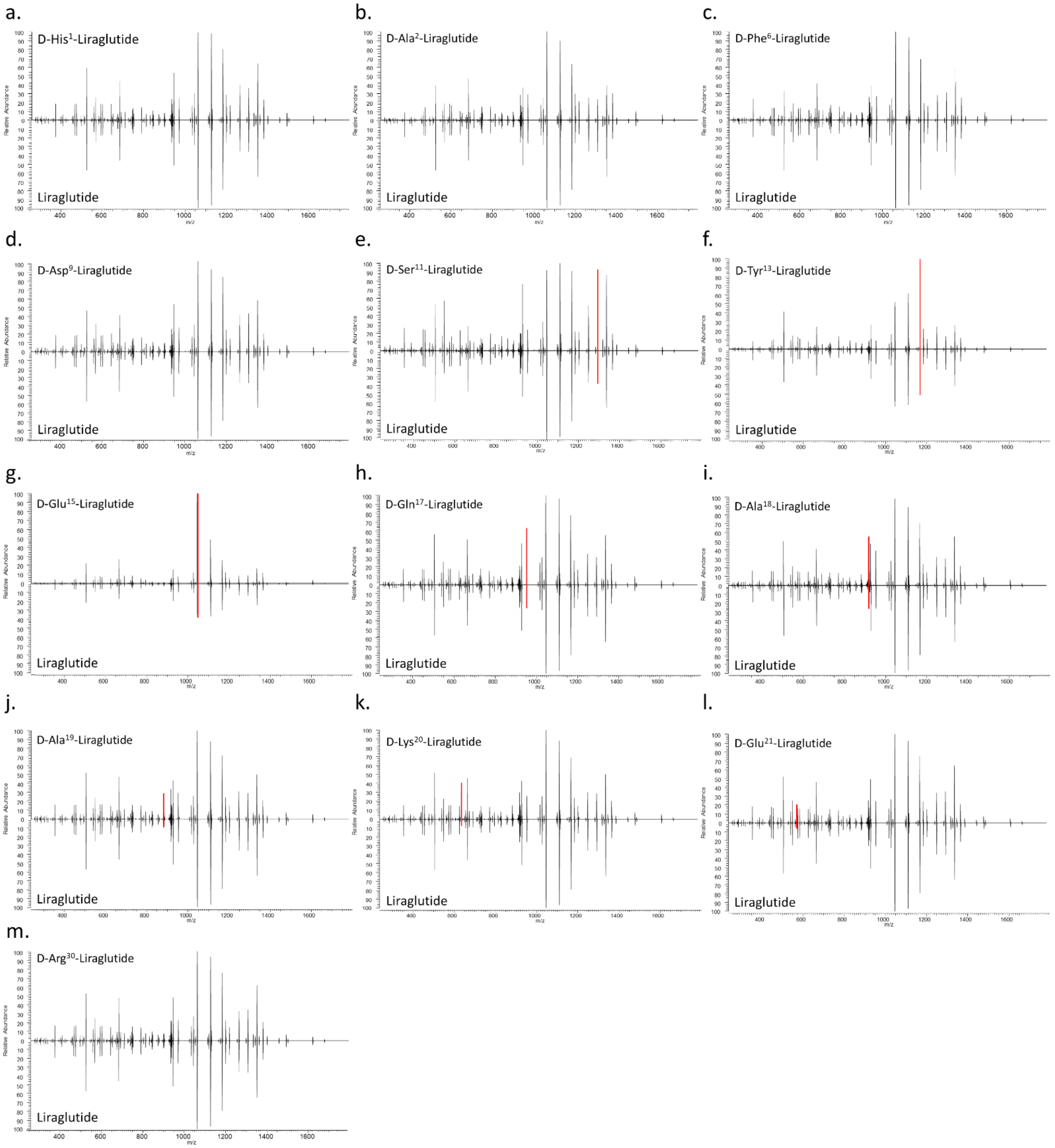

Figure S4. Relationship difference of normalized fragment ion intensity and types of ions in 14 DAACPs, only doubly charged y ion showed significant difference. a. D-His[1]-Liraglutide, b. D-Ala[2]-Liraglutide, c. D-Phe[6]-Liraglutide, d. D-Asp[9]-Liraglutide, e. D-Ser[11]-Liraglutide, f. D-Tyr[13]-Liraglutide, g. D-Glu[15]-Liraglutide, h. D-Gln[17]-Liraglutide, i. D-Ala[18]-Liraglutide, j. D-Ala[19]-Liraglutide, k. D-Lys[20]-Liraglutide, l. D-Glu[21]-Liraglutide, m. D-Arg[30]-Liraglutide.

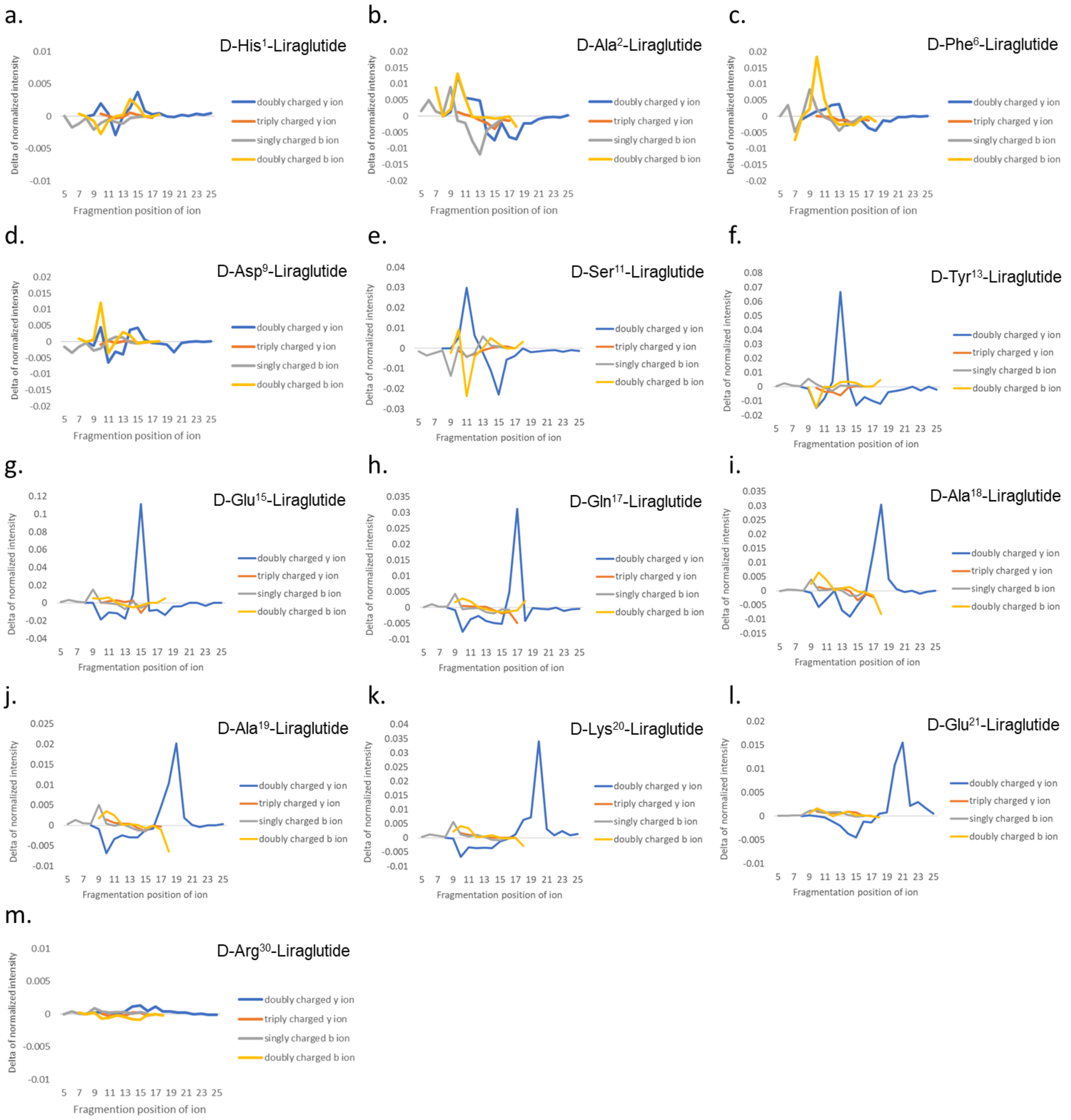

Figure S5. Relationship between difference of normalized doubly charged y ion intensity from triply charged precursor ions and site of D-amino acid.

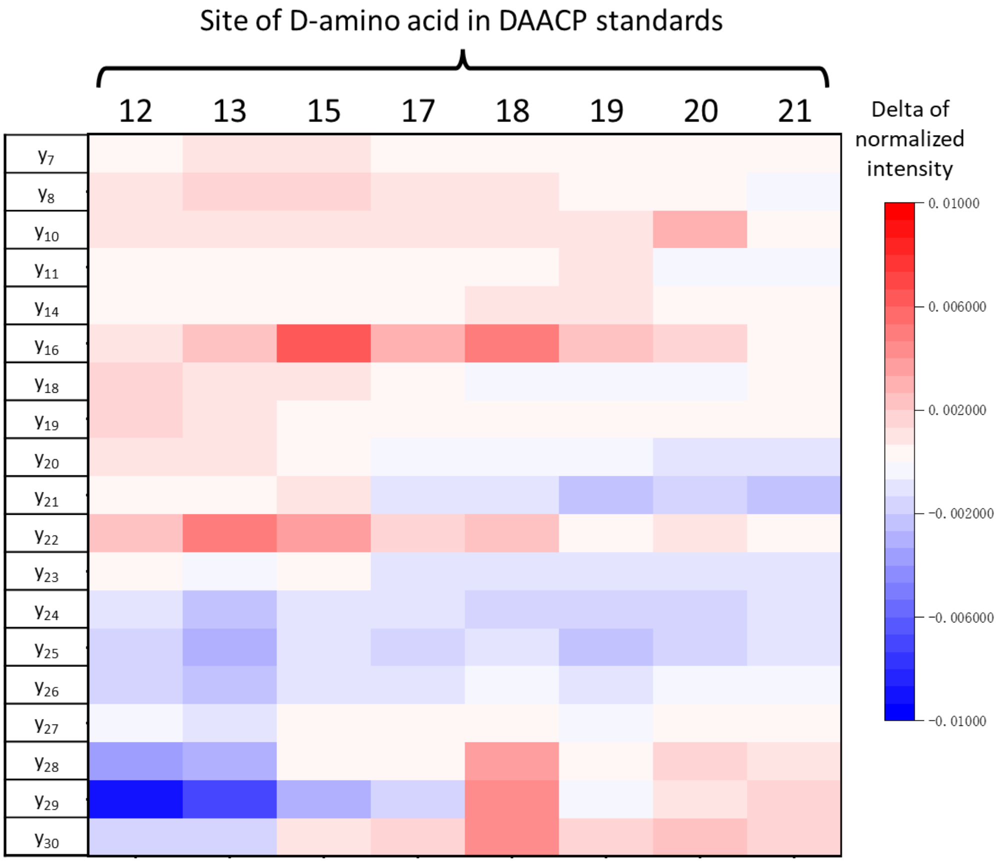

Supplement: Supplementary file 1 [file ijms-25-01379-s001.zip › ijms-2789054-supplementary.pdf]
